# Evaluation of salivary calcium, phosphorus and alkaline phosphatase concentrations before and after the first phase of periodontal treatment in patients with chronic periodontitis

**DOI:** 10.34172/japid.2020.012

**Published:** 2019-10-24

**Authors:** Jaber Yaghini, Samaneh Khashei, Zohreh Afshari, Ahmad Mogharehabed

**Affiliations:** ^1^Dental Implants Research Center, Department of Periodontics, Dental Research Institute, Faculty of Dentistry, Isfahan University of Medical Sciences, Isfahan, Iran; ^2^Private practice, Isfahan, Iran; ^3^Dental student’s research committee, Department of Periodontics, Faculty of dentistry, Isfahan University of Medical Sciences, Isfahan, Iran

**Keywords:** Alkaline phosphatase, Calcium, Periodontal disease, Phosphorus, Saliva

## Abstract

**Background:**

Evaluation of salivary biomarkers is a non-invasive, convenient, and economical method for diagnosing many diseases. Evidence shows that salivary biomarkers and periodontal disease might be correlated. This study was conducted to evaluate phase I periodontal therapy’s effect on salivary concentrations of calcium, phosphorous, and alkaline phosphatase (ALP).

**Methods:**

In this descriptive, analytical study, 16 patients were selected from those referred to the Department of Oral Medicine, Faculty of Dentistry, Isfahan University of Medical Sciences, using convenience sampling. Salivary samples were collected using the drooling method. The salivary concentrations of calcium, phosphorous, and ALP were measured immediately after saliva collection, before the first phase of periodontal therapy and one month later, using a colorimetric assay. The data were analyzed with SPSS using paired t-test. P<0.05 was considered statistically significant.

**Results:**

The salivary concentrations of calcium, phosphorous, and ALP were 6.68, 20.57, and 48.31 mg/dL, respectively, before and 7.15, 22.51, and 40.37 mg/dL, respectively, after phase I periodontal therapy. There were no significant differences between the salivary levels of calcium, phosphorous, and ALP before and after phase I periodontal therapy (P>0.05).

**Conclusion:**

This study revealed that the salivary concentrations of calcium, phosphorous, and ALP remained relatively unchanged after phase I periodontal therapy.

## Introduction

Periodontitis is the inflammatory response of the periodontium characterized by the inflammation of tooth-supporting structures, progressive attachment loss, and bone loss. Periodontal disease is one of the most common causes of tooth loss. Although pathogenic microorganisms are imperative for the initiation of periodontal disease, this would not be enough for periodontal destruction.^1,2^ The development of periodontal disease, like many other chronic diseases, might be influenced by some other conditions that affect the host response to bacterial challenge.^3^

Patil et al^4^ discussed that salivary analysis has some advantages over the analysis of other body fluids. The salivary analysis is an easy-to-use, noninvasive diagnostic method that causes less discomfort for patients (compared with blood tests). Saliva contains almost every ingredient of the serum; thus, the salivary analysis might be an ideal alternative to serum analysis. Several authors have discussed that salivary analysis can be helpful in the diagnosis of some diseases, especially those affecting the oral cavity.^5^

During the periodontal disease development, some enzymes are released from the dead or dying cells of the periodontium, polymorphonuclear leukocytes, inflammatory cells, and epithelial and connective tissue cells of the affected area.^6^ Jabali et al^7^ reported the optimal efficacy of salivary enzyme analysis for the evaluation of the response to periodontal therapy.

Evidence shows that salivary biomarkers and periodontal disease might be correlated.^8-10^ Alkaline phosphatase (ALP), released from polymorphonuclear cells, osteoblasts, and periodontal ligament osteoblasts, is an intracellular enzyme with a bone-dependent metabolism.^11^ Increased activity of ALP might result from active, destructive processes in the alveolar bone, which can be an indicator of severe periodontal disease.^12^ A review article emphasized the importance of evaluating salivary ALP in the diagnosis of periodontal disease.^13^ Calcium and phosphorous are easily absorbed by the dental plaque, leading to calculus formation and deterioration of the periodontal disease.^14^ Although periodontal disease used to be detected only through routine dental examinations, new studies have highlighted the importance of instant screening for periodontal disease. It has been reported that periodontal disease can play a significant role in developing cardiovascular diseases, cerebrovascular conditions, and preterm labor. As a result, periodontal disease screening can be effective in preventing periodontal disease, improvement of oral health, and increasing the quality of life.^5^

Periodontal therapy would decrease gingival inflammation, the number of subgingival black-pigmented bacteria, and the total number of motile organisms.^15^ Since the evaluation of salivary biomarkers is a non-invasive, relatively simple, and economical method to diagnose some diseases, this study was conducted to evaluate the effect of periodontal therapy on salivary levels of calcium, phosphorous, and ALP.

## Methods

This descriptive, analytical study was carried out in the Oral Disease Department of Isfahan Dental School. The patients were selected from scaling and root planing candidates, who had generalized moderate chronic periodontitis using convenience sampling, based on inclusion and exclusion criteria. The inclusion criteria consisted of (1) having at least 15 teeth except for third molars, (2) no history of periodontal treatment during the past six months, and (3) an age range of 20‒55 years. The exclusion criteria consisted of (1) systemic diseases affecting the periodontium or the immune system, (2) chronic use of corticosteroids or immunosuppressive medications, (3) a history of taking antibiotics during the past six months, (7) being under treatment for anxiety disorders, (8) taking medications affecting the salivary glands, such as anti-histamines and tricyclic anti-depressants, (9) taking anti-hypertensive medications, diuretics or other psychotropic medications, (10) orthodontic treatment during the study, (11) pregnancy, (12) breastfeeding, and (13) pulpal pain during the study.

The study protocol conformed to the ethical guidelines of the 1975 Declaration of Helsinki as revised in 2008. Finally, the patients were enrolled after obtaining their informed consent.

Based on the American Academy of Periodontology definition, periodontitis is defined as having at least two interdental areas with attachment loss of >4 mm in two separate teeth, or PD of >5 mm at the interdental area of two separate teeth.^16^ PD was categorized as mild (1‒2 mm), moderate (3‒4 mm), and severe (>5 mm).^17^ Clinical examination was performed by the same examiner using Williams probe ® (Williams probe, Hu-Friedy, USA), and clinical parameters, including PD, plaque index (PI), and bleeding on probing (BOP) were recorded.

The mean PD was assessed in four areas (midbuccal, midlingual, mesial, and distal) of each tooth. The PI of four tooth surfaces was recorded using a standard probe according to the Sillness and Loe plaque index: (0) no plaque, (1) a film of plaque adhering to the free gingival margin and adjacent area of the tooth, which might be recognized by running a probe across the tooth surface, (2) moderate accumulation of soft deposits in the gingival pocket, or on the tooth and gingival margin, which might be seen with the naked eye, and (3) abundant soft matter within the gingival pocket and/or on the tooth and gingival margin. The scores of all the tooth surfaces were summed up and divided by 4 to achieve the mean score for each tooth. The PI was calculated by adding the mean scores of the teeth divided by the total number of the examined teeth. PI<0.1: the absence of plaque (which is achievable through excellent oral hygiene), 0.1<PI<1: moderate plaque accumulation, 2.1<PI<3 considerable plaque accumulation.^16,18^

BOP was measured according to the Ainamo and Bay index, and the percentage of bleeding areas was calculated.^19^

The salivary samples were collected using the drooling method.^1^ The patients were asked not to brush their teeth and not eat or drink for at least one hour before sampling (to prevent saliva contamination). They were requested to sit on a dental chair in an upright position for 30‒60 minutes and rinse their mouth with water immediately before sampling. They were then asked to gently bend their head and frequently drain their saliva collected for five minutes into a 15-mL graded test tube. The test tube was equipped with a funnel for more convenience. The patients were asked not to swallow their saliva or stimulate its secretion through head and face movements during the saliva collection. The volume of the collected unstimulated saliva was reported in milliliters per minute (mL/min). The collected salivary samples were preserved at 24°C. After collecting the samples, they were centrifuged at 3500 rpm according to the instructions provided in the Pars Azmoon kit® (Pars Azmoon, Tehran, Iran), and the most transparent liquid which was accumulated on the top was collected. Auto-analyzer 3000 BT device was used to measure the salivary concentrations of calcium, phosphorous, and ALP using the colorimetric assay. The function of this device was similar to that of an optical-absorption spectrophotometer. The salivary level of calcium was measured using methylthymol blue. In the colorimetric assay, calcium would produce color in the presence of methylthymol blue; the intensity of the produced color is proportional to the number of calcium ions present in the environment. Phosphorous was measured using the UV method, in which the number of phosphorous complexes generated by ammonium molybdate was calculated. The level of ALP was measured via the kinetic method. ALP affects the colorless substrate, 4-nitro phenyl phosphate, converting it to the yellow 4-nitro phenyl. Alterations in optical absorption are proportional to the ALP activity.

One month after phase I periodontal therapy (comprising of supragingival and subgingival scaling and root planing, and oral hygiene instructions), the samples were collected again to measure the salivary levels of calcium, phosphorous, and ALP for the second time. The collected data were statistically analyzed using SPSS with paired t-test. P-value<0.05 was considered significant.

## Results

**Figure 1:**
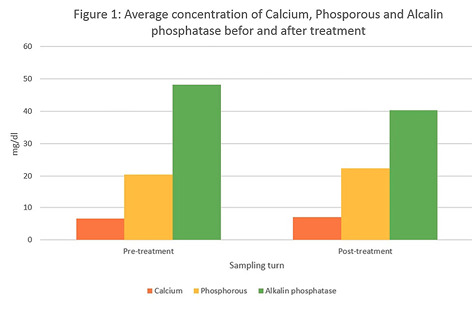
Mean salivary concentrations of calcium, phosphorus, and alkaline phosphatase before and after treatment.

This study evaluated the mean salivary concentrations of calcium, phosphorous, and ALP in patients with chronic periodontitis before and after phase I periodontal therapy. The data obtained from 16 patients were tabulated according to the variables and analyzed using t-test. The mean age of patients was 45 years. Unstimulated salivary samples were collected from all the patients and analyzed. Comparison of the salivary level of calcium, phosphorus, and ALP revealed that the mean concentrations of these biomarkers were 6.68, 20.57, and 48.31 mg/dL, respectively, before and 7.15, 22.51, and 40.37 mg/dL, respectively, one month after phase I periodontal therapy (Figure 1).

Differences in the values before and after phase I periodontal therapy were not statistically significant (P=0.472 for calcium, P=0.542 for phosphorous, and P=0.306 for ALP).

## Discussion

Periodontitis is a multi-factorial disease, and dental plaque is its chief etiologic factor; however, local, systemic, and environmental factors can affect the patients’ response and influence the severity and extent of the disease. Saliva contains numerous enzymes and ions, such as calcium, phosphorous, and ALP.^2,20^

Several authors have reported that salivary analysis might help diagnose several diseases, especially conditions that affect the oral cavity. Several salivary ingredients, such as enzymes, immunoglobulins, hormones, inflammatory cells, and volatile components, have been evaluated as diagnostic markers.^5,21^ The salivary markers widely studied for the diagnosis of periodontal disease include a variety of proteins, ions, and enzymes.^22^

Considering the necessity of screening and early diagnosis of periodontal disease, and since measurement of salivary biomarkers is a non-invasive, relatively simple, and cost-effective method for the diagnosis of some diseases,^23^ this study evaluated the effect of phase I periodontal therapy on salivary concentrations of calcium, phosphorous, and ALP.

No significant differences were found between the mean salivary concentrations of calcium, phosphorous, and ALP before and after phase I periodontal therapy. Other studies conducted on this topic have reported controversial results. Vivek et al^24^ evaluated the relationship between salivary levels of calcium and chronic periodontitis in 40 patients. They reported that patients with chronic periodontitis had higher salivary levels of calcium. In another study conducted by Wong et al,^25^ dental plaques of patients who voluntarily refrained from oral hygiene measures for 24 hours were evaluated. The results showed that calcium plays a role in calculus and plaque formation.

Mohammad et al^26^ evaluated the relationship of chronic periodontitis and the activity of acid phosphatase, and ALP enzymes in the saliva of 60 patients. The results showed that the activity of both evaluated enzymes decreased gradually after scaling and root planing. Inconsistencies between the findings of this study and those of Mohammad et al might be due to different sample sizes or sample collection time.

Totan et al^27^ examined several salivary enzymes in patients with periodontal disease; they found no significant difference in ALP and alanine transaminase activity among patients with/without periodontitis.

It should be noted that the definite diagnosis of periodontal disease, and evaluation of its initiation or progression, or the treatment response is based on clinical examination. An experienced and well-educated dentist should evaluate periodontal indices; however, evaluating some of these indices could be invasive and not cost-effective as a screening method. Therefore, using para-clinical methods, such as laboratory tests as an adjunct to the clinical examination, could be effective in some cases.

This study aimed to evaluate the effect of periodontal therapy and assess the consistency of clinical and para-clinical findings by analyzing the salivary biomarkers, which is a simple and non-invasive method. Although there are significant changes in salivary factors during periodontal disease, the assessment of most of them is not cost-effective. Measuring the salivary concentrations of calcium, phosphorous, and ALP is relatively simple and economical. According to the findings of this study, although the salivary levels of calcium, phosphorous, and ALP did not change considerably after nonsurgical periodontal therapy compared with baseline, these minor changes can be measured to evaluate the periodontal condition and treatment response. A definite conclusion regarding this topic requires comprehensive studies with a larger sample size. In addition, other factors that can affect periodontal disease need to be measured and evaluated. The patients’ diet should be controlled, and new samples should be collected after completion of periodontal therapy.^28^

## Conclusion

This study’s results showed no significant correlation between periodontal disease and salivary levels of calcium, phosphorous, and ALP. As a result, the measurement of the salivary levels of these biomarkers is not recommended for periodontal screening, nor surveillance of the efficacy of periodontal therapy.

## Authors’ Contributions

JY: conceptualization, methodology, definition of intellectual content, manuscript preparation, manuscript preparation, manuscript editing, manuscript review

SK: conceptualization, methodology, clinical studies, data analysis, manuscript editing, manuscript review

ZA: conceptualization, methodology, literature search, manuscript preparation, manuscript preparation, manuscript editing, manuscript review

AM: conceptualization, methodology, definition of intellectual content, manuscript preparation, manuscript editing, manuscript review, funding acquisition, supervision

## Competing interests

The authors declare no competing interests.

## Ethics Approval

The study protocol was reviewed and approved by the local Ethics Committee of Isfahan University of Medical Sciences.
